# Association Between Hemoglobin-to-Red Blood Cell Distribution Width Ratio and 30-Day Mortality in Patients with Acute Pancreatitis: Data from MIMIC-III and MIMIC-IV

**DOI:** 10.5152/tjg.2024.24067

**Published:** 2024-08-01

**Authors:** Jihao Xiong, Hongchun Tan, Shanlin Mao, Lingfang Ma, Ke Ma

**Affiliations:** Department of Emergency-Critical Care Medicine, Huashan Hospital, Fudan University, Shanghai, China

**Keywords:** Hemoglobin-to-red blood cell distribution width ratio, mortality, acute pancreatitis, MIMIC

## Abstract

**Background/Aims:**

To investigate the relationship between hemoglobin-to-red blood cell distribution width (RDW) ratio (HRR) and the 30-day mortality risk in acute pancreatitis (AP), and assess the predictive ability of HRR.

**Materials and Methods:**

Data from 2001 to 2019 in the Medical Information Mart for Intensive Care-III/IV (MIMIC-III/IV) were analyzed. The outcome of this retrospective cohort study was 30-day mortality. Hemoglobin-to-RDW ratio (0-24 hours) and HRR (24-48 hours) were divided into 4 groups based on quartiles (Q1, Q2, Q3, and Q4). The predictive effect was evaluated by the *C*-index.

**Results:**

A total of 1736 patients were included, and 30-day mortality occurred in 204 (11.75%) patients. Compared with Q1 of HRR (0-24 hours), Q2 (HR = 0.60, 95% CI : 0.42-0.86), Q3 (HR =0.47, 95% CI : 0.31-0.71), and Q4 (HR = 0.45, 95% CI : 0.29-0.68) of HRR levels reduced the 30-day mortality risk. Hemoglobin-to-RDW ratio (24-48 hours) was consistent with the results of HRR (0-24 hours). For changes in HRR, Q4 for changes in HRR levels (HR = 1.64, 95% CI : 1.09-2.45) increased the 30-day mortality risk. Hemoglobin-to-RDW ratio significantly improved the predictive effect of Sequential Organ Failure Assessment (*C*-index = 0.736) and Bedside Index of Severity in Acute Pancreatitis (*C*-index = 0.704) on 30-day mortality.

**Conclusion:**

Higher HRR levels reduced the 30-day mortality risk in AP and may improve the prediction of other tools.

**Main Points**

- The relationship between the hemoglobin-to-RDW ratio (HRR) and the 30-day mortality risk in acute pancreatitis (AP) patients was analyzed for the first time.
- Decreased HRR levels increased the 30-day mortality risk in AP patients.
- HRR improved the prediction of other tools (e.g., SOFA and BISAP) for mortality in AP patients.

## Introduction

Acute pancreatitis (AP), an unpredictable and possibly deadly disease, is one of the primary causes of hospitalization for gastrointestinal diseases, with around 300 000 emergency visits annually.^1,2^ It has an increasing incidence, with significant morbidity and subsequent mortality.^3-5^ Moderate to severe AP occurs in about 2% of patients, characterized by pancreatic or peripancreatic tissue necrosis or organ failure, with a mortality rate of 20%-40%.^6^ The discovery of simple and accurate indicators to predict the risk of death in AP patients exhibits important clinical significance.

Anemia is a risk factor for poor prognosis in various diseases.^7-9^ Evidence showed that AP patients with anemia had higher severity, a greater incidence of acute kidney injury (AKI), and longer hospital stays compared to patients without anemia. This may be due to organ hypoxia caused by a decrease in blood oxygen-carrying capacity.^10^ However, the predictive effect of hemoglobin levels for the death risk in AP patients is unclear. In addition to anemia, systemic inflammation is an important pathological mechanism that affects the prognosis of AP patients. Red blood cell distribution width (RDW) can be used as an inflammatory marker in the prognostic assessment of cardiovascular disease, cancer, and other diseases.^11,12^ At present, limited small-sample studies reported that elevated RDW levels in AP patients increased the risk of short-term death.^13,14^ Moreover, the high production of cytokines during inflammation limits iron absorption and inhibits red blood cell (RBC) maturation, which further contributes to anemia.^15^ Recently, a novel indicator combining hemoglobin and RDW, i.e., hemoglobin-to-RDW ratio (HRR), which can comprehensively reflect anemia and inflammatory status, has been used for prognostic evaluation in patients with liver cirrhosis, cardiovascular disease, and cancer.^16-19^ Nevertheless, the relationship between HRR and the risk of death in AP patients is unclear.

We intended to analyze the correlation between HRR and the 30-day mortality risk in AP patients, and evaluate the predictive value of HRR with the data from Medical Information Mart for Intensive Care (MIMIC) databases.

## Materials and Methods

### Sources of Data

Data on AP patients were obtained from 2001 to 2019 in the MIMIC-III/IV databases. Medical Information Mart for Intensive Care contains data on patients admitted to the intensive care unit (ICU) at the Beth Israel Deaconess Medical Center: demographic data, vital sign measurements, laboratory results, etc. Medical Information Mart for Intensive Care-III covered data on more than 40 000 patients between 2001 and 2012; MIMIC-IV comprised data on over 300 000 patients from 2008 and 2019 (https://mimic.mit.edu/docs/about/). The requirement of ethical approval for this was waived by the Institutional Review Board of Huashan Hospital, Fudan University because the data was accessed from MIMIC-III/IV (publicly available databases). The need for written informed consent was waived by the Institutional Review Board of Huashan Hospital, Fudan University due to the retrospective nature of the study. 

### Study Population

In this retrospective cohort study, patients diagnosed with AP were included. AP was determined by International Classification of Diseases, Ninth/Tenth Revision (ICD-9/-10) codes (ICD-9: 5770, ICD-10, starting with K85). Those (1) aged < 18 years, (2) without measurement of baseline (0-24 hours after admission) hemoglobin and RDW, (3) without measurement of hemoglobin and RDW measurements at 24-48 hours after admission, or (4) who died within 2 days immediately after admission to the ICU were excluded.

### Data Collection

The primary outcome was 30-day mortality, i.e., deaths occurring within 30 days of the initial ICU admission. HRR = hemoglobin (g/dL)/RDW (%). Hemoglobin-to-RDW ratio was categorized into 4 groups by quartiles: HRR (0-24 hours) [<0.6178 (Q1), 0.6178-0.7568 (Q2), 0.7568-0.8902 (Q3), and ≥ 0.8902 g/dL (Q4)], HRR (24-48 hours) [<0.5832 (Q1), 0.5832-0.6879 (Q2), 0.6879-0.8151 (Q3), and ≥ 0.8151 g/dL (Q4)]. Hemoglobin-to-RDW ratio was also presented as a continuous variable. Changes in HRR within 0-24 hours and 24-48 hours after ICU admission were calculated. Other data collected within 24 hours of ICU admission included age (years), gender, ethnicity (White, Black, other, unknown), mechanical ventilation, vasopressor, renal replacement therapy (RRT), endoscopic retrograde cholangiopancreatography, blood transfusion, congestive heart failure, respiratory failure, AKI, renal failure, liver cirrhosis, sepsis, malignant tumor, hypertension, aplastic anemia, atrial fibrillation, diabetes, oxygen saturation (SpO2), systolic blood pressure (SBP, mm Hg) and diastolic blood pressure (DBP, mm Hg), Sequential Organ Failure Assessment (SOFA), heart rate (bpm), weight (kg), body temperature (°C), mean arterial pressure (MAP, mm Hg), international normalized ratio (INR), platelet (K/μL), hematocrit, white blood cell (WBC, K/μL), prothrombin time (PT, seconds), fasting glucose (mg/dL), blood urea nitrogen (BUN, mg/dL), Simplified Acute Physiology Score II (SAPSII), alanine aminotransferase (ALT, IU/L), Glasgow Coma Score (GCS), anion gap (mEq/L), creatinine (mg/dL), bicarbonate (mEq/L), sodium (mEq/L), Charlson Comorbidity Index (CCI), respiratory rate (bpm), potassium (mEq/L), Systemic Inflammatory Response Syndrome (SIRS), chloride (mEq/L), bilirubin (mg/dL), aspartate aminotransferase (AST, IU/L), calcium (mg/dL), and Bedside Index of Severity in Acute Pancreatitis (BISAP). Information on length of stay (days) and follow-up time (days) was also obtained.

### Follow Up

Follow-up started 24 hours after the initial ICU admission and ended 30 days after the initial ICU admission or at the time of death. Information on in-hospital mortality was obtained through hospital records. Information on out-of-hospital mortality was obtained from the Social Security Administration Death Master File (https://physionet.org/content/mimiciii/1.4/).

### Statistical Analysis

Continuous data with a normal distribution were reported by mean ± standard deviation (mean ± SD), and the *t*-test was applied to make comparisons between groups. Continuous data with skewed distribution were presented as median and interquartile range [M (Q1, Q3)], and were contrasted by the Wilcoxon rank-sum test. Categorical data were shown by the number of cases and proportion [(n (%)], and inter-group comparison was subject to the Chi-square test or Fisher’s exact test. If the proportion of missing values for a variable was 25% or more, the variable would be deleted; if the proportion was less than 25%, the missing values would be filled using the random forest imputation method. Sensitivity analysis was carried out on these variables before and after the imputation. 

The Cox proportional hazards model, also known as the Cox regression model, is a semi-parametric regression model that simultaneously examines the relationship between multiple risk factors and the occurrence and timing of event outcomes, thereby overcoming the shortcomings of the single-factor limitation of simple survival analysis. The univariable Cox regression model was applied to evaluate the association between covariates and the 30-day mortality risk to screen confounding factors. Covariates with *P *< .05 in the univariable Cox regression model were screened by bidirectional stepwise regression for confounders that were eventually adjusted for in the multivariable Cox regression model. Then the association between HRR and 30-day mortality was investigated using univariable and multivariable models. The multivariable model was adjusted for ethnicity, vasopressor, AKI, liver cirrhosis, sepsis, creatinine, bilirubin, SAPSII, CCI, and BISAP. Restricted cubic spline (RCS) is one of the common methods to study non-linear relationships, which can fit the curve relationship of independent variables and realize the correlation analysis between continuous exposure and results. The non-linear relationship between HRR and 30-day mortality in AP was analyzed by the RCS curve. Kaplan–Meier (KM) curves showed the survival of AP patients with different HRR levels, and the log-rank test was used for comparison. Subgroup analysis was conducted in terms of hypertension, diabetes, respiratory failure, AKI, renal failure, liver cirrhosis, sepsis, and malignant tumor to assess the association between HRR and 30-day mortality among different subpopulations. Bootstrap resampling was utilized to measure the performance of different prediction models. The discrimination of prediction models was evaluated by the concordance index (*C*-index). Hazard ratios (HRs) and 95% CIs were estimated.

Data cleaning (including missing value statistics), missing value imputation, and ROC curve visualization were completed by Python 3.9.12 (Python Software Foundation, Del, USA). Sensitivity analysis, difference comparison, and statistical modeling were done using SAS 9.4 (SAS Institute Inc., Cary, NC, USA). The KM survival curve, RCS curve, and forest plot visualization were realized with R version 4.2.1 (R Foundation for Statistical Computing, Vienna, Austria). 

## Results

### Characteristics of Patients

**Figure 1.**
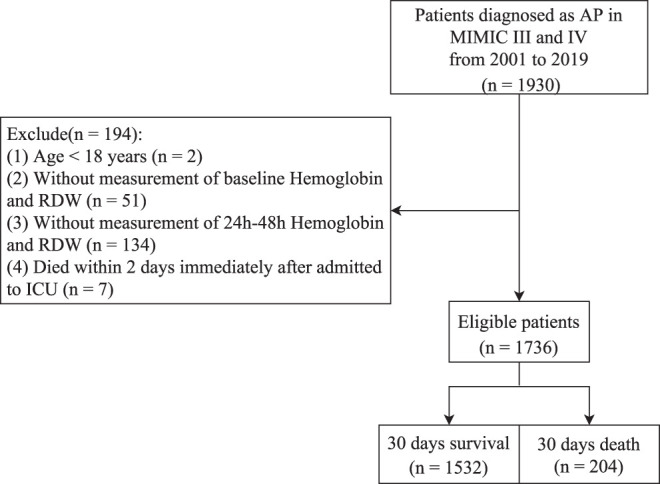
Process of study population selection. AP, acute pancreatitis; ICU, intensive care unit; MIMIC, Medical Information Mart for Intensive Care; RDW, red blood cell distribution width.

From the MIMIC database, a total of 1930 patients diagnosed with AP were enrolled from 2001 to 2019. After excluding patients aged < 18 years (n = 2), those without baseline (0-24 hours) hemoglobin and RDW measurements (n = 51), without hemoglobin and RDW measurements at 24-48 hours after admission (n = 134), and those who died within 2 days of ICU admission (n = 7), 1736 patients were enrolled. Of the included patients, 204 (11.75%) died within 30 days (non-survivors), while 1532 (88.25%) survived for 30 days or more (survivors). The process of study population selection is presented in Figure 1. The mean age was 58.93 years, and men accounted for 57.26% of the total population. Most patients were White. Table 1 lists the detailed characteristics of these AP patients. Survivors were significantly younger than non-survivors (*P *< .001). Moreover, significant differences were also found in ethnicity, mechanical ventilation, vasopressor, RRT, blood transfusion, congestive heart failure, atrial fibrillation, respiratory failure, AKI, liver cirrhosis, sepsis, malignant tumor, SBP, DBP, MAP, body temperature, respiratory rate, WBC, hematocrit, AST, creatinine, BUN, bilirubin, anion gap, bicarbonate, potassium, INR, PT, SOFA, SAPSII, CCI, SIRS, GCS, BISAP, length of stay (days), and follow-up time (all *P *< .05). Survivors had higher hemoglobin levels than non-survivors (11.28 g/dL vs. 10.55 g/dL), and survivors had significantly lower RDW than non-survivors (14.93% vs. 16.31%) (both *P *< .05). The median follow-up time for non-survivors was 12.43 days. 

### Relationship Between HRR and 30-day Mortality

**Figure 2.**
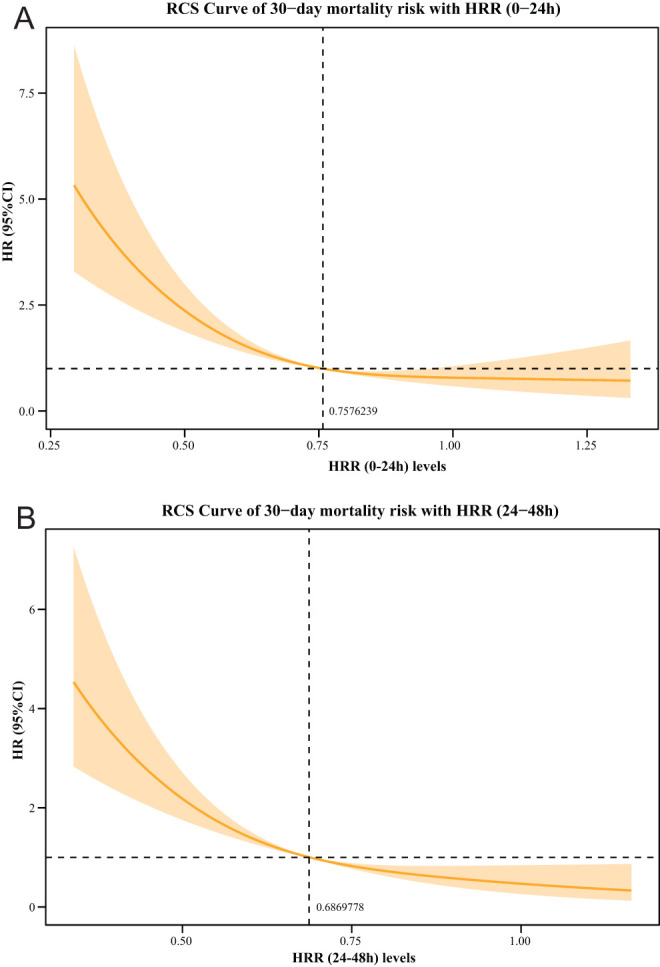
RCS curve for the relationship between HRR and 30-day mortality in AP. (A) HRR (0-24 hours); (B) HRR (24-48 hours). RCS curve, restricted cubic spline curve; HRR, hemoglobin-to-red blood cell distribution width ratio; AP, acute pancreatitis; HR, hazard ratio.

**Figure 3.**
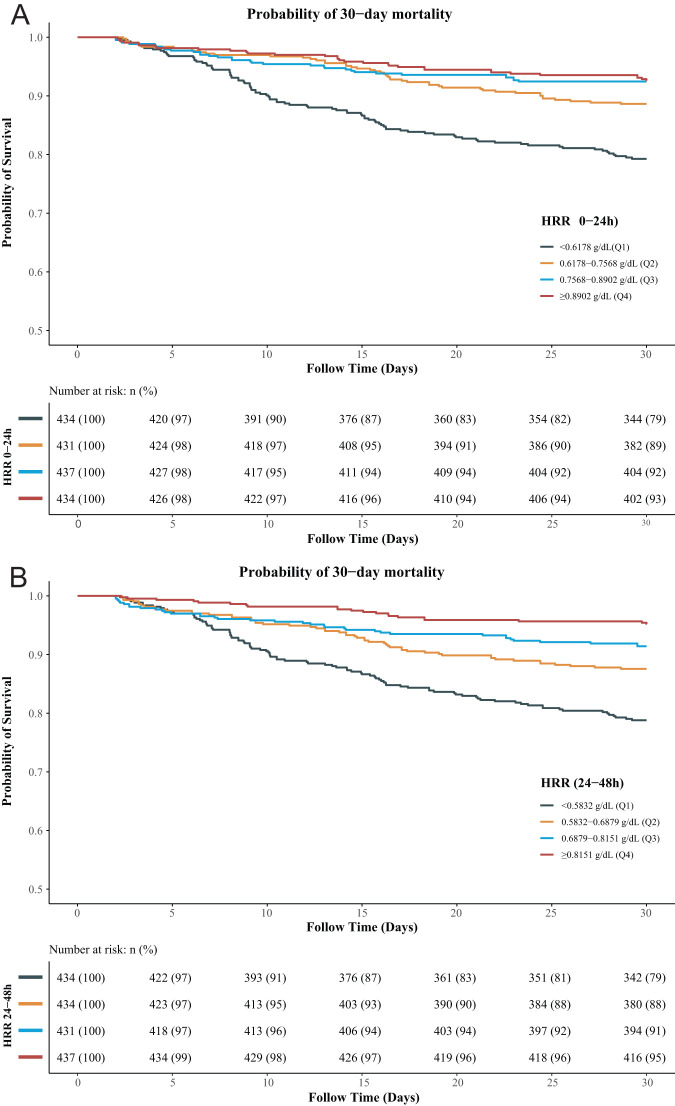
Kaplan–Meier (KM) survival curve for AP patients with different HRR levels. (A) HRR (0-24 hours) and (B) HRR (24-48 hours). HRR, hemoglobin-to-red blood cell distribution width ratio; AP, acute pancreatitis.

Based on the multivariable Cox proportional hazards model, ethnicity, vasopressor, AKI, liver cirrhosis, sepsis, creatinine, bilirubin, SAPSII, CCI, and BISAP were selected as confounding factors to explore the association between HRR and 30-day mortality (Table 2). Table 3 presents the association of HRR (0-24 hours), HRR (24-48 hours), and HRR changes with 30-day mortality. The multivariable analysis demonstrated that compared to Q1 of HRR (0-24 hours), Q2 (HR = 0.60, 95% CI: 0.42-0.86), Q3 (HR = 0.47, 95% CI: 0.31-0.71), and Q4 of HRR levels (HR = 0.45, 95% CI: 0.29-0.68) reduced the 30-day mortality risk. In terms of HRR (24-48 hours), Q2 (HR = 0.61, 95% CI: 0.43-0.86), Q3 (HR = 0.59, 95% CI: 0.40-0.88), and Q4 of HRR (24-48 hours) levels (HR = 0.36, 95% CI: 0.22-0.59) also reduced the 30-day mortality risk compared to those with Q1 of HRR. For changes in HRR, only patients with a change in HRR ≥ 0.0027 (Q4) (HR = 1.64, 95% CI: 1.09-2.45) had a higher risk of 30-day mortality compared with those with a change in HRR <−0.112 (Q1). The RCS curve exhibited a linear relationship of HRR (0-24 hours) and HRR (24-48 hours) with 30-day mortality, and increased HRR (0-24 hours) and HRR (24-48 hours) levels were linked to a lower risk of 30-day mortality (Figure 2). As illustrated by the KM survival curve, patients in the Q2, Q3, and Q4 groups [HRR (0-24 hours) and HRR (24-48 hours)] had a lower risk of 30-day mortality than patients in the Q1 group (all log-rank* P *< .001) (Figure 3). 

### Predictive Ability of HRR for 30-day Mortality

With the bootstrap resampling method, the SOFA combined with HRR (0-24 hours) (*C*-index = 0.736, 95% CI: 0.703-0.769) or HRR (24-48 hours) (*C*-index = 0.736, 95% CI: 0.704-0.769) exhibited a greater discrimination ability than the SOFA alone (*C*-index = 0.711, 95% CI: 0.677-0.745) [HRR (0-24 hours): 0.736 vs. 0.711, *P *= .0176; HRR (24-48 hours): 0.736 vs. 0.711, *P *= .0202], indicating the value of HRR in improving the predictive performance of SOFA (Table 4). In addition, the BISAP combined with HRR (0-24 hours) (*C*-index = 0.704, 95% CI: 0.670-0.737) or HRR (24-48 hours) (*C*-index = 0.713, 95% CI: 0.681-0.745) also presented a greater discrimination ability than the BISAP alone (*C*-index = 0.661, 95% CI: 0.628-0.694) [0.704 vs. 0.661, *P *= .0020; 0.713 vs. 0.661, *P *= .0002].

### Association Between HRR and 30-day Mortality in Subpopulations

**Figure 4.**
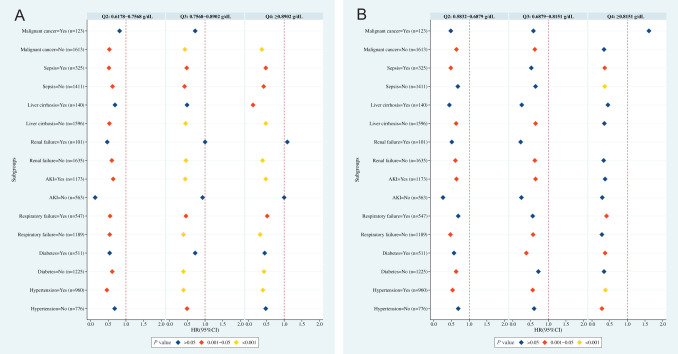
Association between HRR and 30-day mortality in subpopulations. (A) HRR (0-24 hours); (B) HRR (24-48 hours). Q1 [HRR (0-24 hours): <0.6178 g/dL; HRR (24-48 hours): <0.5832 g/dL] acted as a reference. AKI, acute kidney injury; HR, hazard ratio.

Stratified analysis was further conducted in subpopulations with hypertension, diabetes, respiratory failure, AKI, renal failure, liver cirrhosis, sepsis, or malignant tumor (Figure 4).

### Hypertension

In hypertension, patients with Q2, Q3, and Q4 HRR (0-24 hours) and HRR (24-48 hours) had a lower risk of 30-day mortality, as compared with Q1 (all *P *< .05). For patients without hypertension, those with Q3 of HRR (0-24 hours) and Q4 of HRR (24-48 hours) had a lower risk of 30-day mortality than Q1 (*P *< .05).

### Diabetes

For patients with diabetes, the risk of 30-day mortality in the Q2, Q3, and Q4 HRR (0-24 hours) groups was comparable to that in the Q1 group (all *P *> .05), while Q3 and Q4 of HRR (24-48 hours) levels reduced the 30-day mortality risk (vs. Q1, *P *< .05). For patients without diabetes, a reduced 30-day mortality risk was observed in the Q2, Q3, and Q4 of HRR (0-24 hours) groups and the Q2 of HRR (24-48 hours) group (vs. Q1, *P *< .05).

### Respiratory Failure

In respiratory failure, Q2, Q3, and Q4 levels of HRR (0-24 hours) and Q4 levels of HRR (24-48 hours) reduced the 30-day mortality risk compared to Q1 (*P *< .05). In the absence of respiratory failure, the Q2, Q3, and Q4 groups of HRR (0-24 hours) and the Q2 and Q3 groups of HRR (24-48 hours) had a lower risk of 30-day mortality in contrast to Q1 (all *P *< .05).

### Acute Kidney Injury

Among patients with AKI, Q2, Q3, and Q4 levels of HRR (0-24 hours) and Q2 and Q3 levels of HRR (24-48 hours) reduced the 30-day mortality risk (vs. Q1, *P *< .05). In patients without AKI, Q2, Q3, and Q4 levels of HRR (0-24 hours) and HRR (24-48 hours) were not significantly associated with 30-day mortality (vs. Q1, *P *> .05).

### Renal Failure

In patients with renal failure, no significant association was found between HRR and 30-day mortality (*P *> .05). In patients without renal failure, Q2, Q3, and Q4 levels of HRR (0-24 hours) and Q2 and Q3 levels of HRR (24-48 hours) reduced the 30-day mortality risk (vs. Q1, *P *< .05).

### Liver Cirrhosis

Regarding cirrhosis patients, patients in the Q4 of HRR (0-24 hours) group had a lower 30-day mortality risk (vs. Q1, *P *< .05). Non-cirrhosis patients in the Q2, Q3, and Q4 of HRR (0-24 hours) groups, and Q2 and Q3 of HRR (24-48 hours) groups, had a lower risk (vs. Q1, *P *< .05).

### Sepsis

For patients with sepsis, Q2, Q3, and Q4 of HRR levels (0-24 hours), and Q4 of HRR levels (24-48 hours), reduced the 30-day mortality risk (vs. Q1, *P *< .05). Patients without sepsis in the Q2, Q3, and Q4 of HRR (0-24 hours), and Q4 of HRR (24-48 hours) groups also had a significantly decreased 30-day mortality risk (vs. Q1, *P *< .05).

### Malignant Tumor

In the presence of a malignant tumor, the Q2, Q3, and Q4 of HRR (0-24 hours) and HRR (24-48 hours) groups had similar risks of 30-day mortality compared to the Q1 group (*P *> .05). However, in the absence of a malignant tumor, Q2, Q3, and Q4 of HRR levels (0-24 hours), and Q2 and Q3 of HRR levels (24-48 hours), reduced the 30-day mortality risk (vs. Q1, *P *< .05). 

## Discussion

The relationship between HRR and 30-day mortality risk in AP patients was explored, and the value of HRR in predicting 30-day mortality was assessed. The findings demonstrated that decreased HRR (0-24 hours) and HRR (24-48 hours) levels increased the 30-day mortality risk. For changes in HRR at 0-24 and 24-48 hours after admission, only patients in the Q4 group were linked to a higher 30-day mortality risk (vs. Q1). In addition, HRR significantly improved the predictive effect of SOFA and BISAP on 30-day mortality in AP patients.

As an indicator incorporating hemoglobin and RDW, HRR has emerged as a new biomarker associated with mortality in many diseases. High HRR levels at admission were associated with lower long-term mortality in patients with sepsis.^20^ Huang et al^21^ showed that lower HRR was correlated with a greater mortality risk among patients with sepsis-associated encephalopathy. According to another study, HRR was predictive of HBV-related decompensated cirrhosis.^16^ An inverse relationship was observed between HRR and event-free survival in head and neck cancer, as illustrated by Tham et al.’s study.^22^ Our results demonstrated that decreasing HRR levels increased the 30-day mortality risk in AP patients. The potential mechanism of the association between HRR and 30-day mortality is still uncertain. Among individuals with AP, an inflammatory disorder of the pancreas,^23^ RDW, which can reflect the inflammatory state, was observed to be corelated with mortality.^24,25^ As a possible explanation, bone marrow function and iron metabolism may be affected by inflammation. Inflammatory cytokines inhibit the maturation of RBCs, making newer and larger reticulocytes participate in the circulation, which is related to increased RDW.^26^ High oxidative stress also decreases erythrocyte survival, prompting the release of large numbers of prematurely maturing erythrocytes into the peripheral circulation, leading to increased RDW. In addition, inflammation affects erythrocyte membrane glycoproteins and ion channels, leading to changes in erythrocyte morphology.^27,28^ These conditions may be linked to the death risk in AP patients. Furthermore, hemoglobin, the other parameter of HRR, reflects the severity and prognosis of AP patients. Lin et al^29^ found that hypertriglyceridemic AP patients with varying grades of severity had different levels of hemoglobin. A negative association between hemoglobin and mortality in AP was exhibited by another study,^30^ which supported the findings of this study. Anemia may play a role in the correlation between hemoglobin and 30-day mortality. Less oxygen will be transported to major organs under a low level of hemoglobin, resulting in organ hypoxia.^31^ This may promote multi-organ functional dysfunction, which might be associated with short-term mortality. Additionally, hemoglobin levels may be affected by inflammatory responses in several ways, such as inhibiting RBC generation, shortening RBC survival, and reducing the production of erythropoietin.^32^

Of note, this study found that incorporating HRR into the SOFA or BISAP improved the predictive performance of the SOFA or BISAP, which confirmed the predictive ability of HRR in 30-day mortality among AP patients. The SOFA score evaluates organ dysfunction in the ICU, using data on PaO_2_/FiO_2_, platelets, bilirubin, hypotension, Glasgow Coma Scale, and renal function.^33^ Given the significant improvement in the predictive capability of the SOFA by HRR, HRR may be taken into account when assessing the 30-day mortality risk in AP, which may help improve prognoses of AP patients. Besides, in different subpopulations, the relationship between HRR and 30-day mortality varied. No significant association was found in AP patients with malignant tumor and renal failure, which may be attributed to the small sample size included for analysis. The correlation between HRR and the 30-day mortality risk was identified in hypertension, respiratory failure, AKI, liver cirrhosis, and sepsis, which could be related to levels of inflammation.^34-37^

This research first probed the relationship between HRR and the 30-day mortality risk in AP patients and indicated the predictive value of HRR. Hemoglobin-to-RDW ratio, an easily accessible static indicator, serves as a good supplement to the SOFA. With consideration of HRR, clinicians may better predict the prognosis of AP patients and provide timely and personalized treatment strategies for prognosis improvement. Meanwhile, several limitations should be acknowledged. First, due to the retrospective study, selection bias may exist. Second, information on the diet and lifestyle of patients was not collected by MIMIC, and these possible confounding factors were not adjusted for. Third, the findings were obtained based on single-center data, which may limit the generalization of this study. Fourth, although we analyzed the effects of HRR levels and changes in 0-24 hours and 24-48 hours after admission on 30-day mortality, the relationship between changes in HRR over longer periods and patient mortality needs to be further explored. Fifth, the relationship between changes in HRR values after versus before pancreatitis and patient mortality may provide meaningful information, but HRR values before pancreatitis were not available due to database limitations. Sixth, detailed treatment information is an important factor in accurately assessing disease prognosis, but the absence of treatment information in the database may affect the accuracy of our results. 

Decreasing HRR levels increased the 30-day mortality risk and exhibited good predictive ability in 30-day death prediction, indicating that consideration of HRR in clinical prediction of 30-day mortality may facilitate prognosis management in AP. These findings need to be verified in more studies.

## Figures and Tables

**Table 1.** Characteristics of the Included Acute Pancreatitis Patients

Variables	Total (n = 1736)	Survivors (n = 1532)	Non-survivors (n = 204)	*P*
Age, years, mean ± SD	58.93 ± 17.26	57.75 ± 17.20	67.80 ± 15.02	<.001
Gender, n (%)				.741
Female	742 (42.74)	657 (42.89)	85 (41.67)	
Male	994 (57.26)	875 (57.11)	119 (58.33)	
Ethnicity, n (%)				<.001
White	1111 (64.00)	998 (65.14)	113 (55.39)	
Black	186 (10.71)	173 (11.29)	13 (6.37)	
Others	240 (13.82)	204 (13.32)	36 (17.65)	
Unknown	199 (11.46)	157 (10.25)	42 (20.59)	
Ventilation, n (%)				<.001
No	550 (31.68)	515 (33.62)	35 (17.16)	
Yes	1186 (68.32)	1017 (66.38)	169 (82.84)	
Vasopressor, n (%)				<.001
No	1178 (67.86)	1110 (72.45)	68 (33.33)	
Yes	558 (32.14)	422 (27.55)	136 (66.67)	
RRT, n (%)				<.001
No	1559 (89.80)	1403 (91.58)	156 (76.47)	
Yes	177 (10.20)	129 (8.42)	48 (23.53)	
ERCP, n (%)				.501
No	1666 (95.97)	1472 (96.08)	194 (95.10)	
Yes	70 (4.03)	60 (3.92)	10 (4.90)	
Blood transfusion, n (%)				<.001
No	1214 (69.93)	1125 (73.43)	89 (43.63)	
Yes	522 (30.07)	407 (26.57)	115 (56.37)	
Congestive heart failure, n (%)				<.001
No	1345 (77.48)	1215 (79.31)	130 (63.73)	
Yes	391 (22.52)	317 (20.69)	74 (36.27)	
Atrial fibrillation, n (%)				<.001
No	1334 (76.84)	1210 (78.98)	124 (60.78)	
Yes	402 (23.16)	322 (21.02)	80 (39.22)	
Hypertension, n (%)				.168
No	776 (44.70)	694 (45.30)	82 (40.20)	
Yes	960 (55.30)	838 (54.70)	122 (59.80)	
Diabetes, n (%)				.738
No	1225 (70.56)	1079 (70.43)	146 (71.57)	
Yes	511 (29.44)	453 (29.57)	58 (28.43)	
Respiratory failure, n (%)				<.001
No	1189 (68.49)	1085 (70.82)	104 (50.98)	
Yes	547 (31.51)	447 (29.18)	100 (49.02)	
AKI, n (%)				<.001
No	563 (32.43)	549 (35.84)	14 (6.86)	
Yes	1173 (67.57)	983 (64.16)	190 (93.14)	
Renal failure, n (%)				.188
No	1635 (94.18)	1447 (94.45)	188 (92.16)	
Yes	101 (5.82)	85 (5.55)	16 (7.84)	
Liver cirrhosis, n (%)				<.001
No	1596 (91.94)	1434 (93.60)	162 (79.41)	
Yes	140 (8.06)	98 (6.40)	42 (20.59)	
Sepsis, n (%)				<.001
No	1411 (81.28)	1293 (84.40)	118 (57.84)	
Yes	325 (18.72)	239 (15.60)	86 (42.16)	
Malignant tumor, n (%)				<.001
No	1613 (92.91)	1443 (94.19)	170 (83.33)	
Yes	123 (7.09)	89 (5.81)	34 (16.67)	
Aplastic anemia, n (%)				1.000
No	1733 (99.83)	1529 (99.80)	204 (100.00)	
Yes	3 (0.17)	3 (0.20)	0 (0.00)	
SpO_2_, mean ± SD	96.47 ± 3.08	96.49 ± 3.05	96.34 ± 3.31	.514
Systolic blood pressure, mm Hg, mean ± SD	127.62 ± 25.11	128.61 ± 25.00	120.13 ± 24.70	<.001
Diastolic blood pressure, mm Hg, mean ± SD	70.77 ± 18.05	71.44 ± 17.80	65.80 ± 19.15	<.001
MAP, mm Hg, mean ± SD	108.67 ± 20.95	109.55 ± 20.80	102.02 ± 20.97	<.001
Body temperature, °C, mean ± SD	36.91 ± 0.88	36.95 ± 0.86	36.56 ± 0.92	<.001
Heart rate, bpm, mean ± SD	98.40 ± 21.36	98.52 ± 21.29	97.44 ± 21.90	.495
Respiratory rate, bpm, mean ± SD	20.75 ± 6.01	20.64 ± 6.00	21.61 ± 6.08	.030
Weight, kg, mean ± SD	84.28 ± 20.11	84.39 ± 20.03	83.44 ± 20.72	.526
Hemoglobin, g/dL, mean ± SD	11.20 ± 2.28	11.28 ± 2.25	10.55 ± 2.42	<.001
RDW, %, mean ± SD	15.09 ± 2.13	14.93 ± 2.01	16.31 ± 2.57	<.001
Platelet, K/μL, M (Q_1_, Q_3_)	191.00 (132.00, 272.50)	193.00 (135.00, 272.00)	180.00 (107.00, 284.50)	.057
WBC, K/μL, M (Q_1_, Q_3_)	12.50 (8.60, 18.00)	12.20 (8.50, 17.45)	15.30 (9.70, 21.10)	<.001
Hematocrit, mean ± SD	33.51 ± 6.71	33.69 ± 6.62	32.15 ± 7.24	.002
ALT, IU/L, M (Q_1_, Q_3_)	62.00 (30.00, 120.00)	62.00 (30.00, 120.00)	59.12 (26.00, 122.00)	.615
AST, IU/L, M (Q_1_, Q_3_)	65.00 (41.00, 143.00)	63.00 (41.00, 135.50)	76.12 (46.00, 210.00)	.004
Creatinine, mg/dL, M (Q_1_, Q_3_)	1.00 (0.70, 1.80)	1.00 (0.70, 1.70)	1.40 (0.90, 2.40)	<.001
BUN, mg/dL, M (Q_1_, Q_3_)	20.00 (12.00, 35.00)	19.00 (12.00, 33.00)	32.00 (20.00, 52.50)	<.001
Bilirubin, mg/dL, M (Q_1_, Q_3_)	1.20 (0.70, 2.70)	1.12 (0.60, 2.54)	2.03 (0.90, 4.80)	<.001
Calcium, mg/dL, M (Q_1_, Q_3_)	7.80 (7.00, 8.47)	7.80 (7.00, 8.42)	7.80 (7.05, 8.50)	.940
Fasting glucose, mg/dL, M (Q_1_, Q_3_)	126.00 (101.00, 166.50)	125.00 (101.00, 166.00)	129.50 (103.50, 169.50)	.236
Anion gap, mEq/L, mean ± SD	15.72 ± 4.99	15.55 ± 4.92	17.00 ± 5.34	<.001
Bicarbonate, mEq/L, mean ± SD	21.55 ± 5.45	21.72 ± 5.46	20.30 ± 5.28	<.001
Sodium, mEq/L, mean ± SD	138.41 ± 5.54	138.41 ± 5.42	138.45 ± 6.36	.937
Potassium, mEq/L, mean ± SD	4.12 ± 0.86	4.10 ± 0.85	4.26 ± 0.97	.022
Chloride, mEq/L, mean ± SD	104.92 ± 6.98	104.92 ± 6.89	104.95 ± 7.66	.962
INR, M (Q_1_, Q_3_)	1.31 (1.20, 1.50)	1.30 (1.20, 1.40)	1.50 (1.20, 1.85)	<.001
PT, seconds, M (Q_1_, Q_3_)	14.40 (13.30, 16.20)	14.38 (13.30, 15.80)	15.70 (14.10, 19.35)	<.001
SOFA score, M (Q_1_, Q_3_)	5.00 (3.00, 8.00)	5.00 (2.00, 7.00)	9.00 (5.00, 12.00)	<.001
SAPSII, M (Q_1_, Q_3_)	34.00 (25.00, 45.00)	32.00 (23.00, 43.00)	48.50 (39.00, 59.00)	<.001
CCI score, M (Q_1_, Q_3_)	3.00 (1.00, 5.00)	3.00 (1.00, 5.00)	5.00 (3.00, 7.00)	<.001
SIRS score, mean ± SD	2.97 ± 0.92	2.95 ± 0.94	3.18 ± 0.80	<.001
GCS, mean ± SD	13.89 ± 2.32	13.95 ± 2.26	13.45 ± 2.66	.011
BISAP score, M (Q_1_, Q_3_)	2.00 (2.00, 3.00)	2.00 (2.00, 3.00)	3.00 (2.00, 4.00)	<.001
Length of stay, days, M (Q_1_, Q_3_)	3.18 (1.74, 8.75)	3.03 (1.64, 8.10)	6.54 (2.64, 13.28)	<.001
Follow-up time, days, M (Q_1_, Q_3_)	30.00 (30.00, 30.00)	30.00 (30.00, 30.00)	12.43 (6.52, 17.86)	<.001
HRR (0-24 h), g/dL, mean ± SD	0.76 ± 0.20	0.77 ± 0.20	0.67 ± 0.21	<.001
HRR (0-24 h), n (%)				<.001
<0.6178 (Q1)	434 (25.00)	344 (22.45)	90 (44.12)	
0.6178-0.7568 (Q2)	431 (24.83)	382 (24.93)	49 (24.02)	
0.7568-0.8902 (Q3)	437 (25.17)	404 (26.37)	33 (16.18)	
≥0.8902 (Q4)	434 (25.00)	402 (26.24)	32 (15.69)	
HRR (24-48 hours), g/dL, mean ± SD	0.70 ± 0.17	0.71 ± 0.17	0.61 ± 0.16	<.001
HRR (24-48 hours), n (%)				<.001
<0.5832 (Q1)	434 (25.00)	342 (22.32)	92 (45.10)	
0.5832-0.6879 (Q2)	434 (25.00)	380 (24.80)	54 (26.47)	
0.6879-0.8151 (Q3)	431 (24.83)	394 (25.72)	37 (18.14)	
≥0.8151 (Q4)	437 (25.17)	416 (27.15)	21 (10.29)	
HRR change, g/dL, M (Q_1_, Q_3_)	−0.05 (−0.11, 0.00)	−0.05 (−0.11, 0.00)	−0.06 (−0.11, 0.01)	.733
HRR change, n (%)				.082
<−0.112 (Q1)	434 (25.00)	387 (25.26)	47 (23.04)	
−0.112-(−0.0467) (Q2)	433 (24.94)	372 (24.28)	61 (29.90)	
−0.0467 to 0.0027 (Q3)	435 (25.06)	396 (25.85)	39 (19.12)	
≥0.0027 (Q4)	434 (25.00)	377 (24.61)	57 (27.94)	

AKI, acute kidney injury; ALT, alanine aminotransferase; AST, aspartate aminotransferase; BISAP, Bedside Index of Severity in Acute Pancreatitis; BUN, blood urea nitrogen; CCI, Charlson Comorbidity Index; ERCP, endoscopic retrograde cholangiopancreatography; GCS, Glasgow coma score; HRR, hemoglobin-to-red blood cell distribution width ratio; INR, international normalized ratio; M, median; MAP, mean arterial pressure; PT, prothrombin time; Q1, 1st quartile; Q3, 3rd quartile; RDW, red blood cell distribution width; RRT, renal replacement therapy; SpO2, oxygen saturation; SOFA, Sequential Organ Failure Assessment; SAPSII, Simplified Acute Physiology Score II; SIRS, Systemic Inflammatory Response Syndrome; SD, standard deviation; WBC, white blood cell.

**Table 2.** Cox Proportional Hazards Models for Screening Confounding Factors Associated with 30-day Mortality in Acute Pancreatitis

Variables	Univariable Model		Multivariable Model	
	HR (95% CI)	*P*	HR (95% CI)	*P*
Gender				
Female	0.96 (0.73-1.27)	.762		
Male	Ref			
Ethnicity				
White	Ref		Ref	
Black	0.67 (0.38-1.19)	.171	0.75 (0.42-1.35)	.345
Others	1.52 (1.04-2.21)	.029	1.37 (0.94-2.01)	.102
Unknown	2.17 (1.52-3.10)	<.001	2.00 (1.40-2.87)	<.001
Mechanical ventilation				
No	Ref			
Yes	2.32 (1.61-3.34)	<.001		
Vasopressor				
No	Ref		Ref	
Yes	4.71 (3.52-6.30)	<.001	1.80 (1.28-2.52)	<.001
RRT				
No	Ref			
Yes	2.99 (2.16-4.13)	<.001		
ERCP				
No	Ref			
Yes	1.30 (0.69-2.45)	.420		
Blood transfusion				
No	Ref			
Yes	3.27 (2.48-4.32)	<.001		
Congestive heart failure				
No	Ref			
Yes	2.03 (1.53-2.70)	<.001		
Atrial fibrillation				
No	Ref			
Yes	2.24 (1.69-2.97)	<.001		
Hypertension				
No	Ref			
Yes	1.23 (0.93-1.63)	.150		
Diabetes				
No	Ref			
Yes	0.95 (0.70-1.29)	.743		
Respiratory failure				
No	Ref			
Yes	2.18 (1.65-2.86)	<.001		
AKI				
No	Ref		Ref	
Yes	7.01 (4.07-12.06)	<.001	3.16 (1.79-5.59)	<.001
Renal failure				
No	Ref			
Yes	1.36 (0.82-2.27)	.234		
Liver cirrhosis				
No	Ref		Ref	
Yes	3.34 (2.38-4.69)	<.001	1.83 (1.23-2.73)	.003
Sepsis				
No	Ref		Ref	
Yes	3.41 (2.58-4.50)	<.001	1.69 (1.24-2.30)	<.001
Malignant tumor				
No	Ref			
Yes	2.83 (1.96-4.09)	<.001		
Aplastic anemia				
No	Ref			
Yes	–	.978		
SPO_2_	0.98 (0.94-1.03)	.473		
MAP	0.98 (0.98-0.99)	<.001		
Heart rate	1.00 (0.99-1.00)	.492		
Respiratory rate	1.03 (1.01-1.05)	.027		
Weight	1.00 (0.99-1.00)	.533		
Platelet	1.00 (1.00-1.00)	.537		
Hematocrit	0.97 (0.95-0.99)	.002		
ALT	1.00 (1.00-1.00)	.784		
AST	1.00 (1.00-1.00)	.163		
Creatinine	1.07 (1.01-1.14)	.035	0.84 (0.76-0.92)	<.001
Bilirubin	1.08 (1.06-1.09)	<.001	1.04 (1.02-1.07)	<.001
Calcium	1.02 (0.96-1.08)	.588		
Fasting glucose	1.00 (1.00-1.00)	.820		
Anion gap	1.05 (1.03-1.07)	<.001		
INR	1.13 (1.04-1.22)	.003		
PT	1.01 (1.01-1.02)	.004		
SOFA	1.16 (1.13-1.19)	<.001		
SAPSII	1.05 (1.05-1.06)	<.001	1.02 (1.01-1.03)	<.001
CCI	1.24 (1.19-1.30)	<.001	1.20 (1.14-1.26)	<.001
BISAP	1.64 (1.45-1.86)	<.001	1.28 (1.11-1.48)	<.001

In the multivariable model, adjusted variables included ethnicity, vasopressor, AKI, liver cirrhosis, sepsis, creatinine, bilirubin, SAPSII, CCI, BISAP, and HRR as a categorical variable.

AKI, acute kidney injury; ALT, alanine aminotransferase; AP, acute pancreatitis; AST, aspartate aminotransferase; BISAP, Bedside Index of Severity in Acute Pancreatitis; CCI, Charlson Comorbidity Index; ERCP, endoscopic retrograde cholangiopancreatography; HRR, hemoglobin-to-red blood cell distribution width ratio; HR, hazard ratio; INR, international normalized ratio; MAP, mean arterial pressure; PT, prothrombin time; Ref, reference; RRT, renal replacement therapy; SpO2, oxygen saturation; SAPSII, Simplified Acute Physiology Score II; SOFA, Sequential Organ Failure Assessment.

**Table 3.** Association Between Hemoglobin-to-Red Blood Cell Distribution width Ratio and 30-day Mortality in Acute Pancreatitis

Variables	Univariable Model		Multivariable Model	
	HR (95% CI)	*P*	HR (95% CI)	*P*
HRR (0-24 h)				
<0.6178 (Q1)	Ref		Ref	
0.6178-0.7568 (Q2)	0.51 (0.36-0.73)	<.001	0.60 (0.42-0.86)	.005
0.7568-0.8902 (Q3)	0.34 (0.23-0.51)	<.001	0.47 (0.31-0.71)	<.001
≥0.8902 (Q4)	0.33 (0.22-0.49)	<.001	0.45 (0.29-0.68)	<.001
HRR (24-48 h)				
<0.5832 (Q1)	Ref		Ref	
0.5832-0.6879 (Q2)	0.56 (0.40-0.78)	<.001	0.61 (0.43-0.86)	.005
0.6879-0.8151 (Q3)	0.38 (0.26-0.56)	<.001	0.59 (0.40-0.88)	.009
≥0.8151 (Q4)	0.21 (0.13-0.33)	<.001	0.36 (0.22-0.59)	<.001
HRR change				
<−0.112 (Q1)	Ref		Ref	
−0.112--0.0467 (Q2)	1.31 (0.89-1.91)	.165	1.30 (0.88-1.91)	.183
−0.0467-0.0027 (Q3)	0.82 (0.53-1.25)	.350	1.04 (0.67-1.61)	.860
≥0.0027 (Q4)	1.23 (0.83-1.81)	0.299	1.64 (1.09-2.45)	.017

The multivariable model was adjusted for ethnicity, vasopressor, AKI, liver cirrhosis, sepsis, creatinine, bilirubin, SAPSII, CCI, and BISAP.

AP, acute pancreatitis; AKI, acute kidney injury; BISAP, Bedside Index of Severity in Acute Pancreatitis; CCI, Charlson Comorbidity Index; HR, hazard ratio; HRR, hemoglobin-to-red blood cell distribution width ratio; Ref, reference; SAPSII, Simplified Acute Physiology Score II.

**Table 4.** Predictive Performance of SOFA Alone and SOFA Combined with Hemoglobin-to-Red Blood Cell Distribution width Ratio for 30-day Mortality in Acute Pancreatitis

Variables	Concordance (95% CI)	*χ* ^2^	*P*
SOFA	0.711 (0.677-0.745)	Ref	
HRR (0-24 hours)+SOFA	0.736 (0.703-0.769)	5.64	.0176
HRR (24-48 hours)+SOFA	0.736 (0.704-0.769)	5.39	.0202
BISAP	0.661 (0.628-0.694)	Ref	
HRR (0-24 hours)+BISAP	0.704 (0.670-0.737)	9.57	.0020
HRR (24-48 hours)+BISAP	0.713 (0.681-0.745)	14.11	.0002

AP, acute pancreatitis; BISAP, Bedside Index of Severity in Acute Pancreatitis; HRR, hemoglobin-to-red blood cell distribution width ratio; HR, hazard ratio; Ref, reference; SOFA, Sequential Organ Failure Assessment.
